# Recent Advances in Macroporous Hydrogels for Cell Behavior and Tissue Engineering

**DOI:** 10.3390/gels8100606

**Published:** 2022-09-21

**Authors:** Yuan Ma, Xinhui Wang, Ting Su, Feng Lu, Qiang Chang, Jianhua Gao

**Affiliations:** Department of Plastic and Cosmetic Surgery, Nanfang Hospital, Southern Medical University, 1838 Guangzhou North Road, Guangzhou 510515, China

**Keywords:** macroporous hydrogels, cell behaviors, biochemical cues, matrix mechanics, tissue engineering

## Abstract

Hydrogels have been extensively used as scaffolds in tissue engineering for cell adhesion, proliferation, migration, and differentiation because of their high-water content and biocompatibility similarity to the extracellular matrix. However, submicron or nanosized pore networks within hydrogels severely limit cell survival and tissue regeneration. In recent years, the application of macroporous hydrogels in tissue engineering has received considerable attention. The macroporous structure not only facilitates nutrient transportation and metabolite discharge but also provides more space for cell behavior and tissue formation. Several strategies for creating and functionalizing macroporous hydrogels have been reported. This review began with an overview of the advantages and challenges of macroporous hydrogels in the regulation of cellular behavior. In addition, advanced methods for the preparation of macroporous hydrogels to modulate cellular behavior were discussed. Finally, future research in related fields was discussed.

## 1. Introduction

The extracellular matrix (ECM) is a complex network of proteins, proteoglycans, and glycosaminoglycans secreted by cells. Collagens are major components of the ECM, which are primarily responsible for tissue integrity, elasticity, and stability [1]. Proteoglycans and glycosaminoglycans (such as heparan sulfate, heparin, chondroitin sulfate, keratan sulfate, dermatan sulfate, and hyaluronic acid) are another major class of macromolecules that constitute the core of the ECM network, which promote ECM water retention and stress resistance [2]. Other abundant supramolecular assemblies include adhesion molecules (such as laminin, fibronectin, and tenascin), which support cell binding to substrates [3,4]. In general, ECM not only provides adhesion sites and structures for cells but also supports bioactive cues for regulating cell activity and organ function. During natural tissue regeneration, ECM tolerates complex external or internal stimuli. However, the self-healing ability of tissues often declines to owe to trauma and aging [5]. Tissue engineering, developed in the late 20th century, is a strategy that uses scaffolds to bind cells or growth factors to restore and establish normal tissue and organ functions. In organ donation shortages, ECM-analog scaffolds are the preferred choice for repairing and replacement of damaged tissues [6,7].

Hydrogels are hydrophilic polymer networks formed by physical or chemical crosslinking. Biomimetic modification of the hydrogel polymeric network can mimic the biochemical and physical properties of the ECM, enabling cell loading and repair of damaged tissue. The selection of precursors with good biocompatibility to prepare hydrogels is a prerequisite for successful applications, such as biological components [8,9,10,11] or inert polymers [12,13]. Excellent water retention capacity is the most notable feature of hydrogels, which lays the foundation for mimicking the softness and water content of ECM [14,15]. In addition, the mechanical properties of the hydrogels are adjusted in different ways to accommodate the ECM of different tissue types, such as monomer concentration [16,17,18], crosslink density [19,20,21,22], hydrophilicity [23], and copolymerization [24]. The good permeability of hydrogels is the basis for cell infiltration and material exchange. However, the pore size of conventional hydrogels is usually nanoscale, and the resulting physical limitations are not conducive to the clinical translation of hydrogels in tissue engineering, such as (1) restricted supply of nutrients and oxygen as well as excretion of cellular metabolites; (2) difficulty in seeding cells or cells appearing round in nanopore hydrogels; and (3) hard integration between regenerated tissue and hydrogels.

To remove the limitation of pore size on cell behavior, various strategies for fabricating structured macroporous hydrogels have been developed to obtain sufficient pore size [25]. It is worth noting that some inherent challenges still need to be addressed before they can be applied to cell encapsulation. First, some preparation strategies have factors that are not conducive to cell survival, such as toxic solvents [26] and high osmotic pressure [27]. Second, the development of injectable macroporous hydrogels. Compared to structural macroporous hydrogels, injectable hydrogels can deliver and concentrate large numbers of functional cells to desired locations while avoiding implantation surgery and potential re-surgery risks [28]. Third, there is a lack of factors that control cell behavior. Cell fates are directed by soluble factors and/or biochemical ligands in the hydrogel. Fourth, their relatively weak mechanical properties. The introduction of macropores generally reduces the total solids content of the hydrogel, and its stiffness and fatigue resistance further decrease with increasing pore size [29,30]. Fifth, tissue-like biomimetic structures and physical hierarchies in tissues are important for the regulation of cell behavior. However, most techniques for preparing macroporous hydrogels (except for 3D printing and spinning techniques) produce hydrogels which possess random and chaotic internal pores. The lack of a topology that mimics natural tissue limits its role in the encapsulation of certain cells, such as muscle cells [31,32] and nerve cells [33].

**Figure 1:**
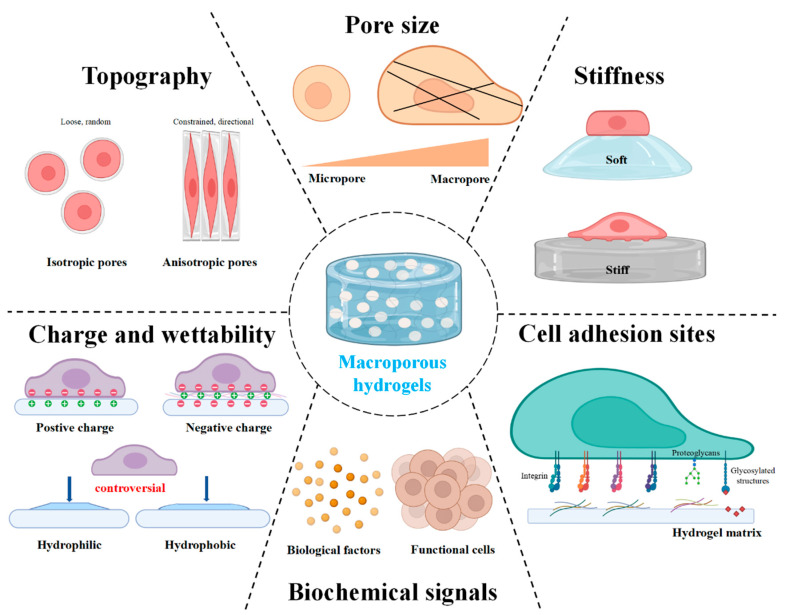
Designing macroporous hydrogels to modulate major factors in cell behavior. The Figure is made with biorender (https://biorender.com/, accessed on 31 July 2022).

In recent years, a detailed review of macroporous hydrogels has been reported. For example, French et al. have focused on structured macroporous hydrogels and discussed their fabrication, characterization, and opportunities [25]. In addition, Fan et al. have cited evidence that macroporous hydrogels enhance anchorage-dependent and anchorage-independent cell viability [34]. Furthermore, Thakar et al. have summarized in detail the functionalization means of macroporous hydrogels in biomedical applications, including antibodies, natural polymers, drug molecules, and protein factors [35]. Regarding tissue engineering applications, Li et. al. have discussed, in detail, the properties of hydrogels for tissue including compositions, mechanical properties, topography, and geometry [36]. However, in the past five years, the preparation strategy of macroporous hydrogels based on tissue engineering has been updated. Moreover, there are still no articles that specifically focus on the complex and dynamic interactions between macroporous hydrogels and cells. Understanding the molecular mechanisms and modifications of macroporous hydrogels that regulate cellular behavior can guide researchers to design more rational products. In our review, we aimed to identify the application of macroporous hydrogels in the regulation of cell behavior and tissue engineering, focusing on research progress based on the above challenges. We focus on new fabrication strategies for macroporous hydrogels in tissue engineering as well as review their molecular mechanisms and improved ways to modulate cellular behavior (Figure 1). The remaining challenges and prospects in the field are also discussed to stimulate and promote further research in this important area.

## 2. Preparation Technology of Macroporous Hydrogels

### 2.1. Sacrifice Templating

#### 2.1.1. Porogen Templating

**Figure 2:**
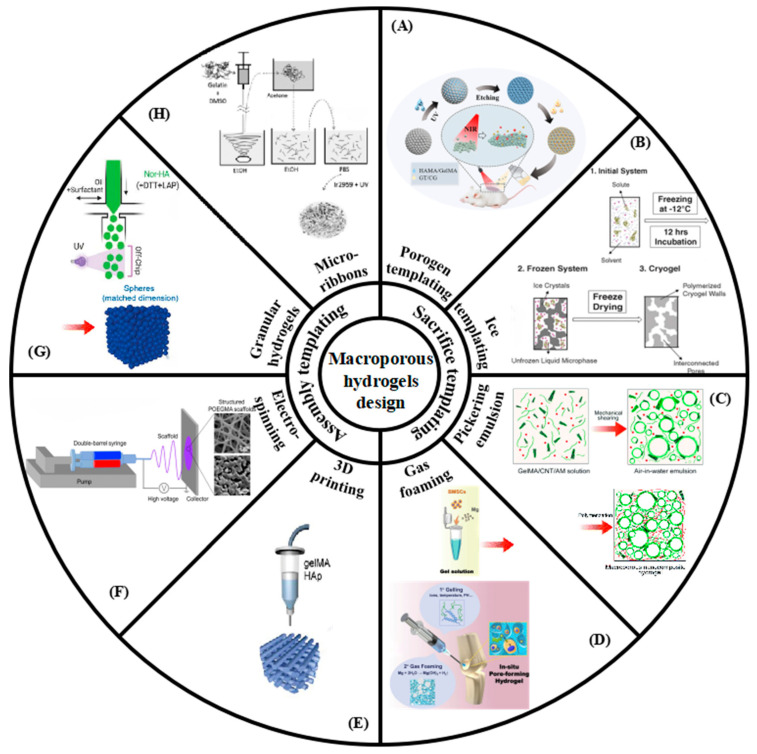
New progress in the preparation of macroporous hydrogels. (**A**) Porogen templating. Reprinted with permission from [171], Copyright 2022, American Chemical Society. (**B**) Cryogel. Reprinted with permission from [172], Copyright 2021, Elsevier. (**C**) Pickering emulsion. Reprinted with permission from [173], Copyright 2020, Elsevier. (**D**) Gas foaming. Reprinted with permission from [68], Copyright 2020, Elsevier. (**E**) 3D printing. Reprinted with permission from [174], Copyright 2021, Elsevier. (**F**) Electrospinning. Reprinted with permission from [94], Copyright Elsevier. (**G**) Granular hydrogel. Reprinted with permission from [100], Copyright 2021, Wiley. (**H**) Microribbon. Reprinted with permission from [166], Copyright 2012, Wiley.

Porogen templating is a conventional method for the preparation of macroporous hydrogels (Figure 2). Usually, solid porogens of a certain size are added to the hydrogel precursor solution and removed later to form large pores in the hydrogels. Macroporous hydrogel polymers suitable for porogen preparation generally include natural, synthetic, and combined natural-synthetic (Table 1). In other words, the choice of porogen usually depends on the type of hydrogel polymer. The selection of the porogens needs two necessary conditions: (1) It is inert and does not react with the hydrogel polymer. (2) It is compatible with the gel formation method of the hydrogel [37].

Since the development of porogen, inexpensive and simple salt templating (such as sodium chloride and sodium carbonate) is the most used porogen in the past. However, it suffers from the problem of hyperosmotic solutions affecting cell survival. Conventional spherical porogens (such as paraffin) increase the pore orientation and degree of interconnection, but require the introduction of additional organic solvents, and are difficult to remove completely. In recent years, many cytocompatible hydrogel porogens have been developed, as well as leaching methods, which are usually divided into water degradation, enzymatic degradation, and dynamic reversible covalent bonds. For example, the macromer poly(ethylene glycol)–norbornene–dopamine (PEGNB-Dopa) is prepared from tandem droplet-microfluidics and thiol–norbornene photopolymerization, which rapidly dissolves in water and releases encapsulated cells in situ, independent of any external triggers or changes in environmental conditions (such as pH and temperature) [110]. Poly (ethylene glycol) (PEG) porogens were developed using a simple microfluidic technique and a Michael-type addition reaction between 4-arm PEG-Ac and DTBA (a dithiol cross-linker). Increasing the proportion of serum in the medium can accelerate the degradation of PEG microspheres, whereas porogen size and cell encapsulation can slow this process [111]. The most significant advantage of hydrogel porogens is that functional factors and cells can be pre-packaged in porogens. Drug and cell loading in conventional macroporous scaffolds are mostly achieved by direct infiltration, which may result in uneven distribution, low efficiency, and long cycle times. The use of hydrogel porogens allows for the in situ release of substances in the macropores for better distribution. For example, gelatin porogens loaded with QK peptides (a de novo engineered VEGF mimicking peptide) can be embedded into an alginate precursor. Gelatin degradation not only produces macroporous structures to enhance the migration and aggregation of stem cells but also continuously releases QK peptides to promote angiogenesis [112].

In the past, the lack of efficiently connected pores and regular topology were the main challenges of porogen technology in the preparation of macroporous hydrogels. In recent years, the inverse opal structure can solve this problem with the following production process: The porogens were arranged in a high degree of order, followed by the addition of a matrix for gelation. Subsequently, porogens were removed as sacrificial templates, resulting in macroporous hydrogels with uniform pore sizes and high pore interconnectivity. For example, alginates were cross-linked by calcium ions to form microbeads and tightly packed in polytetrafluoroethylene (PTFE) molds with a preselected shape and volume, which then infiltrated the interstitial spaces of the microbeads and formed hydrogels by photo-crosslinking [113]. Alginate microbeads were removed by the addition of an ethylenediaminetetraacetic acid (EDTA) solution at room temperature, resulting in macroporous gelatin hydrogels with interconnected original shapes and sizes. However, high packing of porogens can drastically reduce the mechanical properties of macroporous hydrogels, which usually require further enhancement of mechanical properties or are only used for drug delivery [114,115].

Regarding injectable macroporous hydrogels from porogen templates, it is often difficult to form many macropores within small hydrogel microbeads while maintaining their structural stability because of high water content. In addition, the weak macroporous structure is easily destroyed during the processing of microbeads, such as during the washing steps. Recently, the preparation of macroporous hydrogel microbeads with high pore connectivity and mechanical stability by adding functional nanoparticles has become feasible. For example, injectable macroporous alginate microbeads and iron gel microbeads loaded with iron oxide nanoparticles have been prepared, which exhibited good mechanical stability and were stable during needle injection. The increased loading of large biomolecules owing to the macroporosity of the microbeads and their large reversible volumetric deformation response to the external magnetic field enabled their potential for use in the on-demand delivery of drugs of assorted sizes by magnetic actuation [116].

#### 2.1.2. Cryogel

Cryogel is one of the most popular choices for the preparation of macroporous hydrogels due to its simplicity and environmental friendliness, which is formed by the polymer/monomer precursors in a solvent at a low temperature. During the gelation process, ice crystals that act as porogens form inside the hydrogel and are easily removed by thawing or freeze-drying to form interconnected pores. Nucleation theory is the basic principle governing the formation of cryogel pore size and porosity: lower freezing temperatures have faster freezing rates, and their increased nucleation sites lead to the formation of larger numbers of smaller ice crystals. Conversely, as the freezing temperature increases, the pore size in the cryogels also increases, and its distribution becomes looser [117,118]. Lowering the polymer concentration also creates larger pore sizes. Overall, the selection of milder polymer concentrations and freezing temperatures resulted in interconnected macroporous cryogel networks.

From the material properties point of view, cryogels in recent years have mainly focused on the arrangement of pores and the improvement of mechanical properties. Unidirectional freezing is a common method to impart regular pore channels to cryogels. Ice crystals grow in the polymer solution in a set direction during freezing. When polymers are cross-linked and melted, the removed ice crystals form interconnected anisotropic macro-channels within cryogels [119,120,121,122]. Anisotropic cryogels supported better cell migration and tissue infiltration than cryogels with randomly distributed pores [31,123]. Interestingly, Jiang et al. used ice crystals to grow from the periphery to the center of the hydrogel to form a radial topology [124]. Radial topology supported faster cell migration and tissue repair when compared to anisotropic topology in bone tissue engineering. Notably, anisotropic cryogels are structurally compatible with muscles or tendons, but the simple structure cannot provide sufficient mechanical strength [121,125,126]. Various methods have been used to further improve the stretchability and fatigue resistance of cryogels, such as composite [127], hybrid [52], mineralized [128], double network [129], or crosslinking [126]. However, the limited applicability and lifting effect makes the preparation of tough cryogels challenging. In the past year, two simple and universal methods provide new insights into the preparation of fatigue-resistant cryogels. The aggregated state of polymers in cryogels can be further concentrated by combining directional freeze casting and subsequent annealing [121] or salting out [130] treatments, resulting in tough hydrogels. The simplicity, breadth, and reproducibility of these approaches expands the application of cryogels in tissue engineering, such as tendons and ligaments.

The advantages of cryogels include additive-free, economical, efficient, and controllable pore parameters. However, the gel-forming environment at sub-zero temperatures has the potential to impair cell viability, which limits some application of pre-encapsulated functional cells to make cryogels in tissue engineering [131,132]. Recently, Muuray et al. summarized the key factors of cell damage during freezing: (1) The formation of ice crystals in the ECM creates an osmotic gradient in the cell membrane, which leads to cell dehydration. (2) Concentration of cryoprotectants at low temperatures leads to osmotic shock or toxic damage to cells [133]. Common cell cryoprotectants include dimethyl sulfoxide and glycerol. However, they still cause damage to cells and are not suitable for some tissue engineering applications, such as human embryonic stem cells [134]. In addition, cryoprotectants have potential adverse effects on patients, including tonic-clonic seizure and cardiac arrest [135]. Many advanced cell cryopreservation agents have been developed, including vitrification agents, ice recrystallization inhibitors, macromolecular cryoprotectants, ice nucleators, cell encapsulation, intracellular CPA delivery, and the modulation of biochemical pathways [133]. Understanding and utilizing more advanced cell cryopreservation technology will help to expand the application effect and scope of cryogel in tissue engineering.

#### 2.1.3. Pickering Emulsion

Macroporous hydrogels derived from bicontinuous emulsion templates are prepared based on the principle that the oil phase is completely dispersed and immiscible in the water phase. Emulsions are pore templates for hydrophobic phases, which are generally inert and able to miscible with aqueous phases [136]. Based on the above conditions, the hydrophilic phases are suitable for almost any biocompatible hydrogel polymers. However, emulsifier-free emulsions are thermodynamically unstable systems due to the high interfacial area of dispersed droplets [137]. Therefore, it is necessary to add appropriate emulsifiers or stabilizers. High internal phase emulsions (HIPEs) are commonly used emulsion templates, which are colloidal systems with high internal phase volume fractions (φ ≥ 74%) [138]. Traditional HIPEs are formed based on mechanisms such as interfacial film formation [139] or steric hindrance [140]. Most conventional HIPEs are not suitable for tissue engineering due to high toxicity and instability due to the need to add surfactant stabilizers. Therefore, it is necessary to develop new stabilizers. Pickering HIPEs stabilized by particles have received increasing attention. These particulate stabilizers have good and stable properties including two-phase wettability, particle size, shape, surface morphology, and charge [141]. The traditional methods of preparing Pickering emulsions are homogenization and sonication, but the dispersion and size of the particles cannot be controlled. Several simple and controllable emerging technologies have emerged in recent years, including microfluidics [142] and membrane emulsification [143].

The advantages of Pickering HIPEs include easy processing, scalability, and good pore connectivity. However, the limited architecture and resolution makes this method lack a certain topography. Recently, Yao et al. have prepared hierarchical scaffolds with multi-scale pore structures based on 3D printing of the pre-crosslinked oil in water-type hydroxyapatite nanoparticle stabilized Pickering HIPEs hydrogel [63]. The precise characteristics of 3D printing technology make up for the morphological defects of Pickering emulsion to a certain extent, making it have better biological activity.

#### 2.1.4. Gas Foaming

Like porogen templating, almost all biocompatible hydrogel polymers are suitable for gas foaming. It is important to have a clear understanding of the gel time as macroporous hydrogels do not form until a gel point occurs after foaming [144]. Conventional gas foaming technology prepares large pores by introducing a foaming agent into hydrogels. The bubbles generated by the degradation of the foaming agent induce the nucleation and growth of pores, and, finally, form porous structures. Carbonates (such as sodium bicarbonate) are the most used blowing agents because they generate carbon dioxide (CO_2_) under acidic conditions [145]. However, the low water solubility and weak biological effects of CO_2_ limit its application in tissue engineering.

Recent studies have reported the development of a series of foaming agents with biological cues based on the biocompatibility of residual gases and degradants. An injectable macroporous hydrogel with active bubbles in situ and in vivo was recently reported. The macroporous structure was prepared by hydrogen formed from magnesium degradation. In addition, cell viability and proliferation were improved in the magnesium pellet group by adding Mg particles directly to the hydrogel solution containing the cells [68]. Bubbles can also be introduced into a polymer precursor solution or during polymerization using a template-free method. For example, high-speed shear treatment was used to mix air into a precursor mixture of macroporous gelatin (GE)/oxidized sodium alginate (OSA)/adipic acid dihydrazide (ADH) hydrogels, resulting in an injectable double-network macroporous GE/OSA/ADH hydrogel [73].

### 2.2. Assembly Template

#### 2.2.1. 3D Printing

Three-dimensional printing is a powerful and controllable method for printing hydrogels through computer-aided design to form complex structures. Three-dimensional printing techniques for hydrogels can be divided into extrusion printing, inkjet printing, stereolithography, and laser-assisted printing [146]. The type of polymer and viscosity required in different technologies also vary: (1) Extrusion printing is suitable for filament or liquid ink printing with high viscosity (6–30 × 107 mPa·s) [147]. Such as collagen [74,75,76], methacrylamide-modified gelatin (GelMA) [77,78,79], decellularized extracellular matrix [80], and poly(ε-caprolactone) [81]. (2) Inkjet printing is suitable for liquid ink printing with low viscosity (3.5–12 mPa·s) and needs to be processed again to improve stable structure [148]. Such as poly-ɛ-lysine/gellan gum [82] and alginate [83]. (3) Stereolithography is suitable for photopolymerizable prepolymers (no viscosity requirement) [149]. Such as GelMA [84,85]. (4) The laser-assisted printing method is suitable for special photopolymer printing with low viscosity (1–300 mPa·s), where the laser beam is used to build complex structures from ink droplets [147]. Such as collagen [86].

The advantage of 3D printed hydrogels is their ability to fabricate complex macro- and microstructures, showing great potential in meeting the mechanical, structural, and biological requirements for tissue regeneration. The limitation of 3D printing for tissue engineering is the choice of the bio-ink. For cost reasons, extrusion printing is currently the most used. However, the selected polymer must undergo shear thinning after extrusion and quickly return to its high viscosity state. Resolution is another challenge for 3D printing. Typically, 3D printed structures are on the millimeter or centimeter scale, requiring means to make finer micron structures. Recently, Luo et al. realized a 3D printing-based hydrogel with macropores and fully interconnected microchannels [150]. The mechanical strength of the hydrogels was improved by soaking them in a cross-linking solution for a certain period. The surface of the printed scaffold was cross-linked, whereas the interior of the filament was not cross-linked. Then, an interconnected internal network was generated by removing the gel that is not cross-linked from the printed filaments, in which channel walls with a barrier function endowed the scaffolds with the ability to rapidly perfuse fluids in the microchannels.

#### 2.2.2. Electrospinning

Electrospinning is a technique that uses high pressure to overcome the surface tension of extruded polymer solutions and draw them into nanofibers. The advantages of electrospinning-prepared scaffolds are interconnected pores and microfibrous structures whose pore size and fiber parameters are controllable [151]. Electrospinning techniques require consideration of many parameters, including polymer concentration, solvent, relative humidity, high pressure, collector, working distance, solution viscosity, and flow rate [152]. In addition, hydrogel-based electrospinning must achieve the cooperation of flow extrusion and collection gel. Therefore, the proper gel time and sufficient viscosity of the hydrogel are very important.

The advantage of electrospinning to fabricate scaffolds is the interconnected pore and microfibrous structure with controllable pore size and fiber parameters. Common strategies include tuning machine or polymer solution parameters, but the concomitant increase in fiber diameter is detrimental to the microscopic regulation of cells. In addition, selecting different types of collectors (such as pattern collectors and liquid bath collectors) allows for customized pores and structures [94]. A more cost-effective means is to integrate with other pore-forming technologies. For example, the co-deposition of ice crystals with polymers is achieved by cryo-electrospinning techniques. After the sublimation of ice crystals, large pores are generated in the scaffold, which is more conducive to the migration of chondrocytes [153].

#### 2.2.3. Granular Hydrogels

Granular hydrogels are new macroporous hydrogels prepared by assembling microgel particles. Microgel particles must possess the physical properties of self-assembly, shear thinning, and self-healing [154]. Commonly used polymers include alginate [155], silk [156], gelatin [157], chitosan [158], and hyaluronic acid [158,159,160]. These biopolymers are typically functionalized with multiple reactive groups covalently attached to their chains, such as acrylates or methacrylates [161].

The first step is the prefabrication of injectable microgel particle monomers using techniques such as physical pulverization, oil-in-water/water-in-oil emulsion polymerization, microfluidics, photolithography, and microarray chips. After injection and filling of preformed microgel particles into the damaged site of the tissue, they were assembled into a single stable structure (named granular hydrogels) using a non-toxic interconnection technology, such as covalent crosslinking [162] and cell–particle adhesion [154,163].

Current research focuses on the modification of microgels to achieve functionalized granular hydrogels. Microgels were prefabricated using a microfluidic water-in-oil emulsion approach and peptide modifications, including cell adhesion peptides (RGD) and transglutaminase peptides (K and Q) [164]. Subsequently, the exogenous activating factor XIII (FXIIIa, a natural enzyme that stabilizes thrombi) mediates the annealing reaction between K and Q peptides to generate cell-loaded granular hydrogels. Human dermal fibroblasts (HDF), adipose-derived mesenchymal stem cells (AhMSCs), and bone marrow-derived mesenchymal stem cells (BMhMSCs) showed high viability, better proliferation, and spread in granular hydrogels compared with non-porous hydrogels [164]. Likewise, granular hydrogels showed better integration, faster tissue regeneration, and low immune responses in animal models. The advantage of granular hydrogels is that they have an interconnected macroporous network originating from the spaces between the adjacent microparticle hydrogels. Direct control of the pore structure can be achieved by changing the size (usually the micrometer scale based on the cell size), shape, and random orientation of the microparticle monomers. For example, granular hydrogels with pore sizes ranging from 10 to 35 μm can be constructed using building block sizes ranging from 30 to 150 μm in diameter [164]. Granular hydrogels composed of rod-like particles possess superior pore interconnectivity, porosity anisotropy, and contact-guiding cues compared to spherical particles, which enhance cell invasion [100]. Furthermore, a single granular hydrogel can be assembled from a mixture of microparticle hydrogel monomers with different biochemical cues and physical properties, enabling simultaneous regulation of diverse cellular behaviors and tissue generation in complex internal microenvironments. Recently, Zhang et al. reported a method for the manufacture of hybrid granular hydrogels, where microgels were produced by squeezing methacrylamide-modified hyaluronic acid (MeHA) and 3-aminophenyl boronic acid-modified sodium alginate (SABA) nanoporous hydrogels through steel meshes. Both were crosslinked under physiologically alkaline conditions with ionic bioglass (BG) products, resulting in the in situ assemblies of macroporous scaffolds [165].

#### 2.2.4. Microribbons

Microribbon (μRB) is injectable macroporous hydrogel prepared via wet spinning. After syringe extrusion and cross-linking, macroporous and highly flexible 3D scaffolds are formed to support cell proliferation [166]. The method of making μRBs included wet spinning the pre-solution into microfibers and removing solvent. Then, the microfibers were dried and subsequently modified to allow further crosslinking into 3D scaffolds. The continuous pore structure and relatively low cost are the advantages of μRB. The clinical translation of μRB has focused on cartilage and osteogenic tissue engineering to support different kinds of stem cells [167,168,169,170]. However, there is a risk of carryover from using too much non-cellular benign organic solution. Furthermore, the complex and harsh fabrication steps not only slow down the production of macroporous hydrogels but also limit the choice of hydrogel polymers. For a long time in the past, gelatin was the only polymer of μRB that dissolved in highly viscous dimethyl sulfoxide, and subsequently, was extracted from ethanol.

Recently, Gegg et al. prepared μRB from different polymers (chondroitin sulfate, hyaluronic acid, and polyethylene glycol), which were obtained by the solubilizer dithiothreitol and the extractant 2-propanol as well as methacrylate-based photocrosslinking [109]. These μRBs can be mixed in any ratio and cross-linked in a modular fashion into 3D scaffolds, which play a synergistic role in tissue engineering applications. In addition, the macroporosity and mechanical properties of microribbons can be tuned by changing the wet spinning rate, drying temperature, choice of desiccant, crosslinking level, and microstrip density [108]. Based on the above viewpoints, the development of more polymers and tissue engineering applications will be beneficial to the future development of μRB.

## 3. Biophysical and Biochemical Factors of Macroporous Hydrogels for Cell Behavior

### 3.1. Pore Size

**Figure 3:**
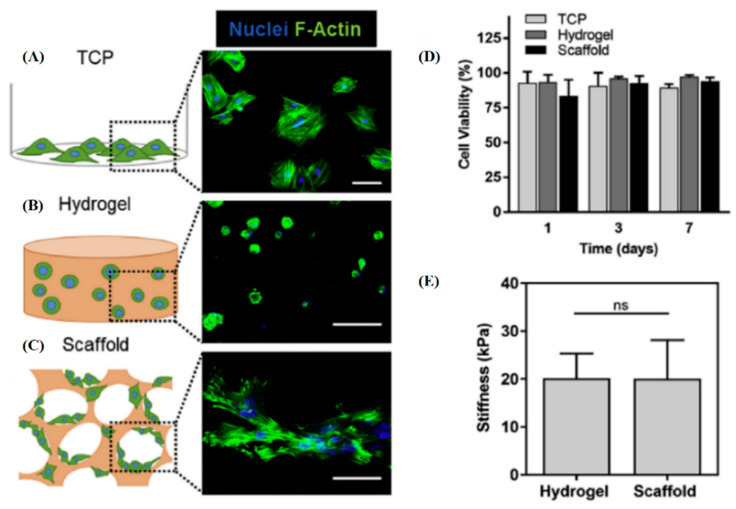
The schematic depiction and corresponding morphological evaluation of MSCs cultured in different substrate microenvironments: (**A**) TCP well plate, (**B**) encapsulated in an alginate hydrogel, and (**C**) seeded on a macroporous alginate scaffold. MSCs were stained with DAPI (nuclei = blue) and Phalloidin (F-Actin = green). Scales (**A**) and (**B**) = 100 μm, and (**C**) = 50 μm. (**D**) MSCs maintained long-term viability on the three substrates after 1, 3, and 7 days. (**E**) Alginate hydrogels and scaffolds showed similar mechanical properties. Reprinted with permission from [179], Copyright 2017, Elsevier.

Pore size is an important parameter affecting cell behavior. On the one hand, hydrogels with sufficient pore size improve cell behavior, benefiting from better diffusion of nutrients and metabolites. On the other hand, pore changes can impose different degrees of geometric constraints on cells, thereby regulating cell behavior and fate according to different molecular signaling pathways. The size and shape of pores in hydrogels determines cell volume and morphology, and the associated molecular expression levels (actin filaments, focal adhesions, nuclear shape, Yes-associated protein (YAP)/transcriptional coactivator with PDZ-binding motif (TAZ) localization, cell contractility, nuclear accumulation of histone deacetylase 3, and lineage selection) has also changed [175]. YAP and TAZ are regulators of single-cell volume, which bind cytoskeletal tension during cell cycle progression [176]. With decreasing pore size, actin and microtubules in hMSCs changed from filamentous to a more dispersed network, and paxillin-rich focal adhesions disappeared [177]. Therefore, sufficient pore size is fundamental for cells to maintain an adherent and spreading morphology. The introduction of interconnected macropores by cryogelation in hyaluronic acid (HA) hydrogels upregulate filamentous actin (F-actin) expression and maintains the spread morphology of hMSCs [178]. Macroporous hydrogels with an average pore size of 120 μm are more favorable for N-cadherin-mediated cell–cell interactions than hydrogels with an average pore size of approximately 5 nm, thereby enhancing the paracrine effect of MSCs (Figure 3) [179].

**Figure 4:**
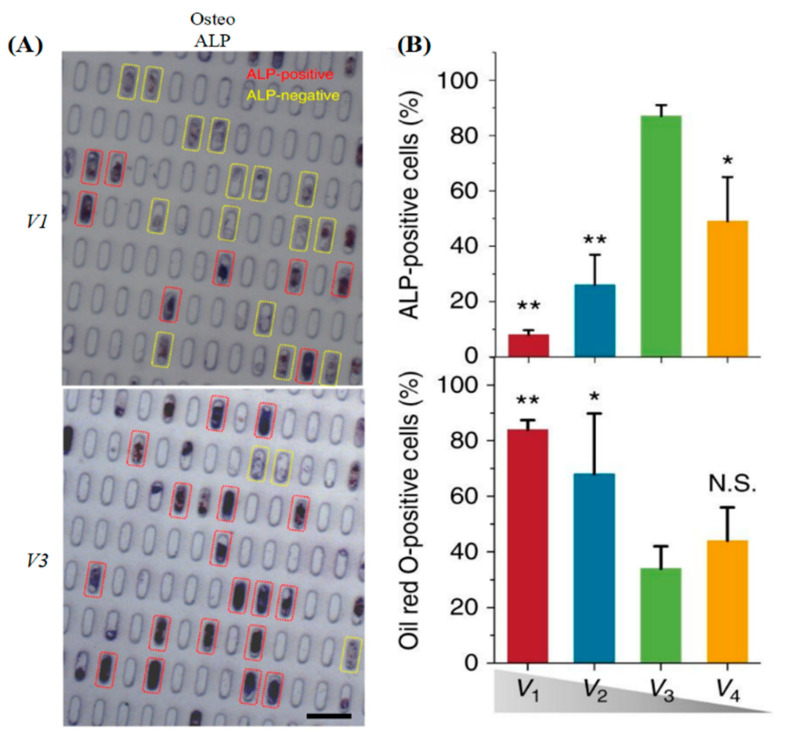
Size affects single hMSC fate. (**A**) Alkaline phosphatase (ALP) staining for cells with different V1 and V3 volumes (V1 > V3). The ALP-positive cells were determined by applying an optimal threshold to the image; ALP intensity above the threshold was determined as ALP positive. (**B**) Quantification of differentiation after 7 days (ALP) and 10 days (Oil Red O) for cells with different volumes. Mean ± s.d., ANOVA one-way analysis followed by Tukey post-hoc test shows significance levels of * *p* < 0.05, ** *p* < 0.01. N.S. no significant difference. Reprinted with permission from [175], Copyright 2017, Springer nature.

In addition, pore size is involved in cell proliferation and viability. Cells require minimal space for survival and mitotic division, and micropores confine and squeeze the nucleus impairing the normal progression of the cell cycle [180]. Mechanosensitive checkpoints in cells can monitor surrounding spatial constraints to control cell cycle progression at the G1-S boundary. Reduced mechanical restraint is accompanied by increased cell volume and activates cell migration and proliferation based on cell cycle reactivation due to MAPK/ERK kinase (MEK) upregulation [181]. Moreover, pore size also significantly affected cell migration. Different pore size constraints alter the relative contributions of cytoskeletal (actin and microtubule) and contractile (myosin II and Rho kinases) machinery to spatial migration [177,182]. Enhancement of GTP-binding proteins Rac 1 activity in the cellular unrestricted channel by preventing α4/paxillin binding to regulate migration, whereas inhibition of Rac1 with α4/paxillin binding in the restricted channel enhances myosin II-driven contractility to regulate migration. In cells lacking α4β1 integrin, the myosin II isoforms MIIA and MIIB are involved in restricted and unrestricted migration, respectively [183]. Unlike single cells, the width of the pore regulates the formation of collective cellular organization migration patterns through cell–cell interactions. As the pore width increases, the migration speed of the cell sheet decreases overall and the pattern changes from a contraction–elongation motion to a large-scale vortex [184]. The pore size in the hydrogel also affects cell differentiation and fate. For example, hMSCs showed differentiation differences in pores of different volumes (The horizontal dimension is 400 µm^2^, and the vertical heights are 23, 12, 9, and 7 µm, denoted as V1, V2, V3, and V4, respectively). Among them, V1 showed adipogenic differentiation, while V3 showed osteogenic differentiation (Figure 4) [175].

Therefore, compared with microporous hydrogels, cells in macroporous hydrogels have a better behavioral regulation ability. For example, the large pore size of 30 ± 6 μm prepared by high internal phase emulsion in 0.5 wt% cellulose nanocrystals (CNCs) functionalized with 2-ureido-4-[1 H]-pyrimidinone motifs (CNC-Upy) hydrogel facilitates the distribution of gelatin and further enhances the adhesion of mouse bone marrow mesenchymal stem cells (mBMSCs) [185]. GelMA/ hydroxyapatite nanowires (HANWs) composite macroporous cryogel seeded at 143.7 ± 32.1 μm rather than 36.4 ± 8.1 μm is more favorable for BMSCs diffusion [186]. The migration distance of BMSCs in the 143.7 μm GelMA/HANW cryogel is also much longer than that in the 36.4 μm hydrogel [186]. Furthermore, 50 μm significantly inhibited the chondrogenic differentiation and gene expression of BMSCs. More than 300 μm provided growth space for BMSCs, but excessive cell proliferation resulted in the reduction of differential gene expression. The introduction of macropores of 372.73 ± 30.15 μm in silk-sericin-based (SS)/GelMA0.33 by 3D printing enabled fibroblasts to possess better adherent morphology and cell interconnection [187]. Three-dimensional bioprinted macroporous GelMA hydrogels were prepared by the introduction and removal of aqueous dextran microdroplets. The optimal pore size obtained by adjusting the ratio of the two is 81.5 ± 30.1 μm, which not only enhances the expansion area and spreading morphology of fibroblasts and myoblasts but is also more conducive to the reconstruction of cartilage tissue [188]. In granular hydrogels formed by in situ annealing of microgel particles to one another, human dermal fibroblasts show good diffusion and the diffusion area increases with a pore size (60–200 μm) [189]. Gelatin macroporous hydrogels with an average diameter of 253 μm support the inward migration of human dermal fibroblasts. In contrast, nanoscale pore-sized gelatin hydrogels restrict cell migration and only grow on the hydrogel surface [190].

**Figure 5:**
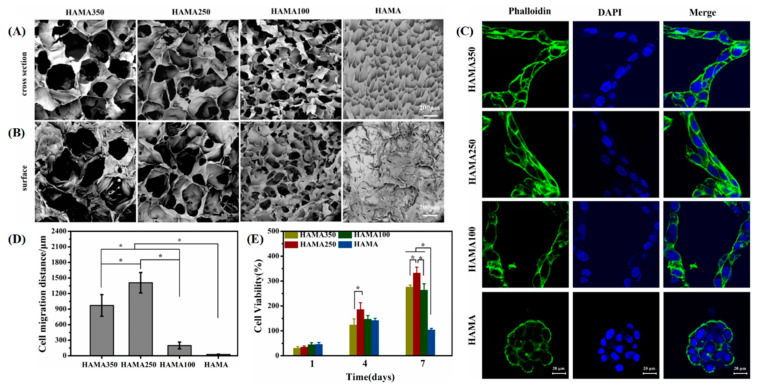
Cell behavior of vascular endothelial cells in hydrogels with different pore sizes. SEM images of cross-section (**A**) and surface (**B**) of HAMA350, HAMA250, HAMA100, and HAMA hydrogels. Scales = 200 μm. (**C**) Representative confocal images of HUVECs on hydrogels after 7 d. The cytoskeleton was stained with iFluorTM 488 Phalloidin (green) and the nucleus was stained with DAPI (blue). Scales = 20 μm. (**D**) Cell 3D migration distances into HAMA350, HAMA250, HAMA100, and HAMA hydrogels after 7 d culture in vitro (* *p* < 0.05). (**E**) Cell proliferation of HUVECs seeded on HAMA350, HAMA250, HAMA100, and HAMA hydrogels after 1, 4, and 7 d by CCK-8 assay (* *p* < 0.05). Reprinted with permission from [194], Copyright 2022, IOP Publishing.

In addition, the pore sizes of macroporous hydrogel preparations also vary by cell and tissue. For example, 100 μm is suitable for the differentiation of the superficial cartilage layer, and 200 μm is suitable for the expression of the middle cartilage layer [191]. Chondrocytes (CCs) were suitable for 100 and 200 μm pore size, BMSCs were suitable for 200 μm pore size, and tendon stem cells (TCs) were suitable for 200 and 300 μm pore size [191]. GE/OSA/ADH macroporous hydrogels with an average diameter of 380 μm are more favorable than those with a diameter of 250 μm for NIH 3T3 cell migration and nutrient transport [73]. Optimal adhesion and spreading of neuronal cells require a pore size of at least 100 μm [192]. Injectable RGD+ hyaluronic acid hydrogels prepared by cryogelation require sufficiently large (50 ± 0.5 μm) and highly interconnected (83.5 ± 2.8%) pores, which are more suitable for chondrocytes to interact with the matrix and maintain spindle-shaped morphology [193]. Compared to 120–150 μm and 300–350 μm pore sizes, macroporous hydrogels with 200–250 μm pore sizes are more favorable for the migration and proliferation of human umbilical vein endothelial cells (HUVECs) (Figure 5) [194]. The optimal pore size for vascularization of hydrogels in vitro and in vivo is different. For example, 200 μm gelatin macroporous hydrogel supports optimal differentiation of endothelial progenitor outgrowth cells (EPOCs) in vitro, while 300 μm pore size hydrogel-loaded EPOCs have a better vascular invasion in vivo [195]. The reason for these gaps may be the difference in stiffness, and the specific mechanism will be explained later.

### 3.2. Stiffness

**Figure 6:**
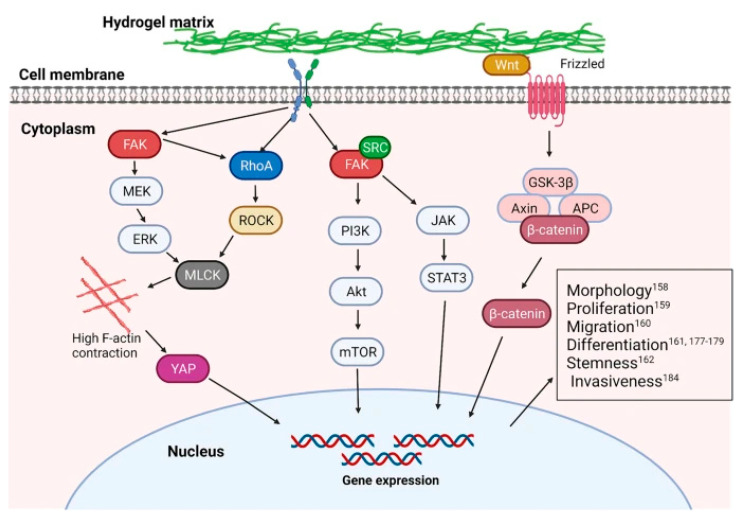
Pathways are involved in the regulation of cellular behavior by hydrogel stiffness. Reprinted with permission from [200], Copyright 2021, Springer nature.

Tissues in both a normal physiological state and tumor tissue in a pathological state have different elastic moduli. Cells are very sensitive to cell adhesion substrate mechanics and different cells require different stiffness to function [196]. For example, the optimal stiffness for migration of embryonic chick forebrain neuronal cells is ~1 kPa, while the optimal stiffness for migration of U251 glioma cells is ~100 kPa [197]. Because cells are in a 3D hierarchy, their different sensors receive signals at different levels. Focal adhesion formation is influenced by microscale stiffness, whereas integrin-mediated signaling is influenced by nanoscale stiffness [198]. In addition, different sensors play different roles in different stiffnesses. For example, Piezo1 is a mechanosensor of macrophage stiffness, and its activity modulates polarization responses [199]. Typical pathways related to stiffness include focal adhesion kinase (FAK) signaling, Ras homolog gene family member A (RhoA) signaling, and the canonical wingless/integrated (Wnt) signaling, which together are involved in the regulation of cellular function [200] (Figure 6).

Likewise, the stiffness of the hydrogel will also affect the morphology and behavior of cells. Cells prefer to grow and express on macroporous hydrogels with moduli similar to those of native tissues. However, macroporous hydrogels generally exhibit lower modulus due to their inherent softness and high porosity. To adapt the stiffness of macroporous hydrogel scaffolds to specific tissue regeneration applications, many approaches have been used to enhance their mechanical properties. Increasing the crosslinking strength is the simplest strategy to reinforce macroporous hydrogels. For example, enhanced genipin crosslinking makes macroporous chitosan hydrogels more suitable for fibroblast adhesion after G’ > 2 kPa [201]. Increasing the sodium hyaluronan/hyaluronic acid crosslinking ratio from 14 to 56 resulted in a 4.4-fold increase in the stiffness of the granular hydrogels (from 222 to 970 Pa) and enhanced HDF diffusion [189]. However, increasing the crosslinking strength is often accompanied by a weakening of the porosity and functionalized molecules, which limits its application to a certain extent. The second method is to increase the cross-linking network. For example, double-cross-linked macroporous hydrogels were fabricated by creating nucleophilic side groups of native fibroin and soaked in hexafluoroisopropanol (HFIP), which exhibited a 20-fold improvement in mechanical properties over single-cross-linked fibroin hydrogels and was more conducive to the formation of larger cell focal adhesions [202]. Increasing the proportion of solids, including polymers and nanoparticles, is another strategy. For example, the addition of hydroxyapatite to polyvinyl alcohol macroporous hydrogels upregulated the modulus, favoring chondrocyte adhesion [203]. Zeolites are microporous materials made of metal cations or organic amine/ammonium cations as templates or structure directing agents, which are commonly used to enhance the mechanical properties of chitosan scaffolds and modulate cellular behavior [204]. For example, a zeolite-A/chitosan macroporous hydrogel with 0.5% zeolite showed higher efficiency in viability and attachment of hBMMSCs and better cell viability than pristine chitosan [205]. Mg ions are involved in normal physiological activities, which are related to cell behavior, bone formation, and healing. MgO nanoparticles (NPs) were introduced into GelMA hydrogels prepared by the Pickering emulsion method, which not only acted as effective emulsion stabilizers but also enhanced the mechanical properties of the hydrogels and mediated the sustained release of Mg^2+^ [67].

### 3.3. Cell Adhesion Sites

#### 3.3.1. ECM

The introduction of ECM molecules into macroporous hydrogels is the most used method for enhancing cell adhesion via integrins. For example, genipin has been used to cross-link collagen with 3D-printed macroporous HA-PVA hydrogels, which showed good chondrocyte adhesion [203]. Covalent cross-linking of laminin to macroporous synthetic polymer hydrogels using poly l-ornithine solution or N-(3-dimethyl aminopropyl)-N-ethyl carbodiimide hydrochloride upregulated stem cell adhesion, including embryonic stem D-3 (ES-D3) mouse embryonic stem cells [206], Lund human mesencephalic (LUHMES), and human embryonic stem cells (hESCs) [207]. Fibronectin (FN) and HA macroporous cryogels enhance the retention of human embryonic kidney cells (HEK 293 cells) by the covalent cross-linker of 1-Ethyl-3-(3-dimethyl aminopropyl) carbodiimide (EDC)/N-hydroxysuccinimide (NHS) [208].

#### 3.3.2. ECM Derivatives

Gelatin is a hydrolyzed derivative of collagen and a key component of human and animal skin tissues. Cross-linking of gelatin with agarose macroporous hydrogels using glutaraldehyde enhances the hydrogel structure and prevents gelatin loss in cell culture [209]. GelMA is usually prepared by reacting the primary amine groups of gelatin with methacrylic anhydride, which is well-immobilized on macroporous hydrogels by photo-crosslinking and supports cell adhesion, including hMSCs [178], hTMSCs, fibroblasts [210], MSCs [211], and mBMSCs [185]. A more promising approach is to simultaneously introduce gelatin and macroporous structures into the hydrogels. For example, gelatin macroporous microstrip hydrogels fabricated by wet spinning can be used for a neatly adherent arrangement of cells [212]. Arginine–glycine–aspartate peptide (RGD) is a peptide derived from fibronectin or collagen, which provides cells with integrin sites and links to integrins that activate cells, thereby allowing cells to adhere to the ECM [213]. Modification of macroporous hydrogels with RGD is widely used, which successfully upregulates MSCs, GBM (which overexpresses the associated αvβ3 and αvβ5 binding integrins) [214], chondrocytes [193], PC12 cells [215], HEK 293 cells [208], and adipose-derived stem cells (ADSCs) [216]. Notably, a small amount of RGD was sufficient to support the adhesion of HUVECs to dextran macroporous hydrogels [217], suggesting that the presence or absence of adhesion molecules affects cell adhesion more than the concentration. Cell migration usually involves alternating processes of old and new adhesions. Lower concentrations of adhesion molecules are not conducive to adhesion formation, whereas higher concentrations of adhesion molecules are not conducive to de-adhesion. Therefore, a moderate concentration of adhesion molecules is more favorable for cell migration within the scaffold. For example, the migration distance of HUVECs inside 0.1% RGD-modified dextran macroporous hydrogels was much longer than that of 1% RGD-modified dextran, which may be related to the fact that lower concentrations of adhesion molecules facilitate cell deadhesion [217].

#### 3.3.3. Other Cell-Responsive Ligands

Blood and its derivatives are important sources of cell-responsive ligands. For example, platelet lysate (PL) contains many proteins and growth factors that regulate cell behavior. Adhesion and spreading of ADSCs are well supported by macroporous cryohydrogels obtained by cross-linking PL with oxidized dextran [55]. Fibrinogens are important proteins involved in the coagulation process. Coating PLGA microribbon (μRB) with fibrinogen not only improves its injectability and in situ cross-linking, but also further supports the encapsulation and rapid adhesion of hMSCs in hydrogels [218]. Lectin is a glycoprotein that can agglutinate red blood cells by specifically binding to glycosylated structures on the cell surface. Macroporous hydrogels were modified with lectin affinity for fructose, the concentration of which was upregulated in proportion to the adhesion area of human alveolar basal epithelial cells (A549). Interestingly, the use of higher affinity sugars can elute lectin to release the cells, so that cell–material adhesion is reversed [219].

### 3.4. Biochemical Signals Release

In addition, chemotaxis is an inherent tropism response of cells to external stimuli. a Macropores enhance the diffusion of adhesion molecules in the hydrogel, and the resulting adhesion signal gradient guides cell migration into the scaffold. For example, the macroporous structure and concentration gradient produced by the degradation of gelatin microspheres together guide the migration of hMSCs into hydrogels [112,194]. Likewise, gelatin-based injectable macroporous hydrogels support the migration of large numbers of cells (mainly corneal epithelial cells) from porcine corneal tissue into the scaffolds [190]. In addition, the concentration gradients of different growth factors/chemical signals are involved in directing specific cells. For example, concentration gradients generated by QK peptides from gelatin microspheres guide the migration of HUVECs from the surrounding environment to macropores [112]. The addition of soy protein or soy peptide powder (the product of soy protein proteolysis) to 3D printed macroporous alginate/gelatin-based hydrogel can guide the migration of HUVECs and blood vessels to the scaffold [188]. Recently, an acoustically responsive scaffold composed of a perfluorooctane phase-shift emulsion loaded with basic fibroblast growth factor (bFGF-C8) and fibrin matrix hydrogels was developed. Vaporization of the bFGF-C8 by adjusting the position of suprathreshold ultrasound, generation of its macroporous structure, and the release of bFGF support the directional migration of fibroblasts in different spatial modes [220]. The introduction of cells into macroporous hydrogels for co-culture also significantly improves migration owing to the secretion of paracrine factors associated with tissue regeneration. For example, the introduction of IL-4-overexpressing MSCs within macroporous μRB scaffolds significantly enhances macrophage migration into the scaffolds and subsequent bone formation [221]. Likewise, the introduction of MSCs into macroporous alginate hydrogels induces a collective migration behavior of myoblasts to the scaffold, which is associated with an increase in paracrine factors produced by the upregulation of N-cadherin, mediating MSCs’ interconnected communication. About proliferation, hydrogels coated with ECM secreted by mesenchymal stem cells, dermal fibroblasts, or osteoblasts are more effective than substrates coated with ECM from human osteosarcoma cells lines. Constitutive motifs with an affinity for fibronectin can regulate integrin binding to switch between proliferation and differentiation by regulating fibronectin conformation [222]. In addition to their natural components, organic polymers also regulate cell proliferation. It has been reported that polymers with appropriate hydrophobicity can promote cell attachment and proliferation in vitro. Surface modification with -COOH groups increases cell proliferation, whereas surfaces modified with -NH groups favor migration rather than proliferation [223]. In addition, coating hydrogels with inorganic components can also be used to modulate cell proliferation. For example, calcium phosphate-coated surfaces exhibit increased cell proliferation compared to uncoated surfaces [224].

### 3.5. Charge and Wettability

Cell adhesion is closely related to the charge of the material, and has also been demonstrated in macroporous hydrogels. For example, macroporous hydrogel-cell interactions were improved by the addition of positive or negative charges, which were proportional to the amount of added charge [225]. Notably, cells are more inclined to rapidly adhere to the positively charged modified macroporous hydrogels. For example, cell adhesion and growth in double macroporous poly (2-hydroxyethyl methacrylate) hydrogels modified with positively charged quaternary ammonium groups are far superior to those of negatively charged groups, doubly charged groups, and unmodified groups [206]. Modified diethyl aminoethyl methacrylate (DEAEMA) ^(+)^ is more suitable for the adhesion of fibroblasts and hMSCs than modified acrylic acid (AA) ^(−)^ and unmodified (none) macroporous Acr-PVA hydrogels [225].

**Figure 7:**
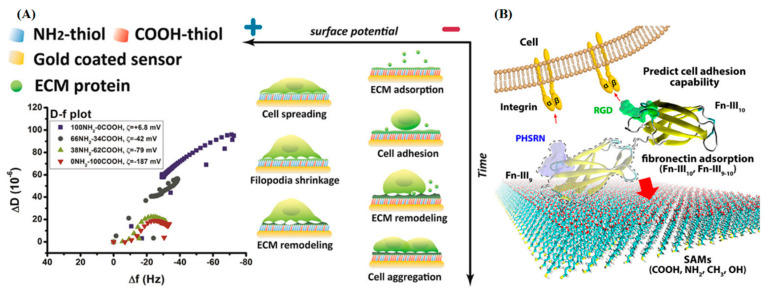
(**A**) The relationship between cell adhesion and ECM matrix surface charge. Reprinted with permission from [226]. Copyright 2017, American Chemical Society. (**B**) Role of the ninth type-III domain of fibronectin in the mediation of cell-binding domain adsorption on surfaces with different chemistries. Reprinted with permission from [227], Copyright 2018, American Chemical Society.

Recently, a mechanism related to the surface charge of cells and materials has been demonstrated (Figure 7). Cells adhere differently to differently charged surfaces. On the one hand, cells can interact directly with positively charged surfaces because of the negatively charged cell membrane. On the other hand, cells cannot directly adhere to negatively charged surfaces. They must undergo an initial electrostatic repulsion and subsequent ECM secretion, culminating in an indirect cell–ECM negatively charged surface interaction. It means that the charge can attract adhesion proteins to the environment to mediate cell adhesion [226]. In addition, charge also affects cell–material interactions by changing the conformation of cell adhesion molecules. Cell-binding domains of adhesion proteins exhibit a more favorable spatial structure and orientation on positively-charged surfaces than on negatively-charged surfaces [227]. It is worth noting that although the cell adhesion morphology in macroporous hydrogels improves after modification of positive charge or laminin alone, the combination of the two reaches an optimal value [192,225]. Therefore, comprehensive consideration of the positive charges and adhesion proteins is a promising strategy for obtaining better cell adhesion.

Wettability (hydrophilic and hydrophobic) is also one of the factors affecting cell behavior. The surface wettability of macroporous hydrogels affects protein adhesion, thereby regulating changes in cellular behavior [228]. However, the specific mechanism by which wettability affects cell behavior is unclear. There are currently two main views, and they are opposed to each other. First, cells from mammals are generally thought to be more inclined to adhere to moderately hydrophilic surfaces. Both superhydrophilic (water contact angle < 5°) and superhydrophobic (water contact angle > 150°) hydrogel surfaces are unfavorable for cell adhesion [229,230]. However, recent studies have shown that superhydrophilic (water contact angle < 5°) and wetted superhydrophobic surfaces (water contact angle from 100 to 140°) are more suitable for cell adhesion [231]. For example, cryo-electrospun poly(ε-caprolactone) (PCL) (CE) encapsulated in macroporous gelatin/heparin cryogel (GH) showed improved hydrophilicity (water contact angle from CE103° to CEGH0°), which supports the attachment of fibroblasts [232]. It should be noted that the effect of wettability on cells is not dominant compared to the adhesion site and stiffness of the hydrogel [41,201]. This may be the reason for the different views between wettability and cells. In conclusion, the influence of wettability on cells needs to exclude the influence of other parameters, which requires further exploration and attention in the future.

### 3.6. Topography

During development, cell shape is regulated and restricted to specific topologies to drive fundamental processes of tissue and organ morphogenesis [233]. Introducing topology into macroporous hydrogels not only induces the differentiation process but also affects specific functions of the resulting cells. For example, macroporous hydrogels can be prepared by embedding chitosan (CS) and sodium alginate (SA)-based polyelectrolyte complexes (PECs) in a poly(acrylamide) (PAM)-crosslinked network, and the presence of a ladder-like fiber topology enhances osteogenesis and cell adhesion [234].

Preparation of different pore shapes is the most common topology in macroporous hydrogels [175]. Among them, anisotropic macroporous hydrogels have attracted considerable attention. The surface or internal pores in the same direction guide cell adhesion and alignment, which is suitable for special tissue engineering, including nerves and muscles. A sacrifice template is the most used method for the preparation of anisotropic macroporous hydrogels. For example, Schwann cells cultured on macroporous polyacrylamide (PAM)-YIGSR peptide hydrogels prepared from PDMS stamps showed more spread and oriented morphology [235] (Table 2). Likewise, both the morphology and focal adhesion of fibroblasts on macroporous PAM hydrogels prepared using polyethylene terephthalate (PET) stamps were aligned parallel to the pore direction, which was attributed to the imposition of the pattern on the cells [236]. A templating method called “hot ice” was previously reported, which prepared anisotropic agarose/gelatin (AG) hydrogel via the seeds-initiated crystallization of NaAc 3H_2_O. The final pore size of the aligned AG gels can vary from 30 to 300 μm, supporting directional migration and alignment of NIH3T3 cells [209]. Macropores from directional freeze–thaw were obtained based on the generation and sublimation of anisotropic ice crystals within the hydrogel at low temperatures. In macroporous RGD–alginate hydrogels with a diameter of 100 ± 15 μm, fibroblasts and hBMSCs were aligned along the pore direction [237]. 

For assembly templates, spinning is used to prepare anisotropic macroporous hydrogels directly. For example, anisotropically aligned alginate fibers can be easily obtained using a high-speed rotating collector in electrospinning. Fibronectin-modified fibers supported the adhesion of hMSCs and controlled their extension in the fiber direction [238]. In addition, pH-dependent electrospun polyacrylonitrile (PAN) hydrogel supports the adhesion of ADSCs and cardiomyocytes along the nanofiber orientation [239]. Compared with ordinary gelatin–MA hydrogels, wet-spinning-prepared aligned μRB macroporous hydrogels support rapid adhesion and uniform arrangement of human bladder-derived smooth muscle cells (SMCs) instead of maintaining circular shapes, which mimic the anisotropy of the muscle alignment structure [212]. The 3D printing technology has received considerable attention in recent years. For example, well-defined mesh thread patterns by 3D printing alginate-based hydrogels help hADMSCs obtain more ordered adhesion and distribution [240]. Recently, an anisotropic hydrogel fabricated using highly versatile ultrasound-assisted biofabrication (UAB) was reported to support the directional adhesion and alignment of human adipose-derived stem cells or chondrocytes. UAB can be used in combination with 3D extrusion printing and stereolithography and has the potential to produce anisotropic macroporous hydrogels [241]. 

**Figure 8:**
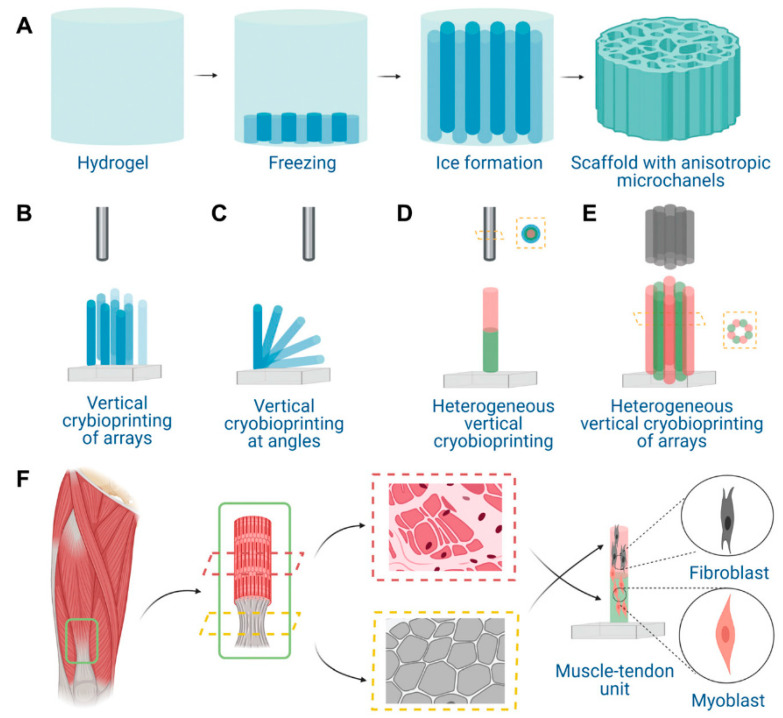
Schematic illustrations of vertical 3D cryo(bio)printing and its application in muscular tissue engineering. (**A**) The GelMA-based hydrogel, when subjected to directional freezing, forms interconnected gradient of anisotropic microchannels along the vertical axis. (**B**) Vertical 3D cryo(bio)printing of hydrogel filament arrays. (**C**) Vertical 3D cryo(bio)printing of hydrogel filaments of different angles. (**D**) Multimaterial vertical 3D cryo(bio)printing of a single hydrogel filament. (**E**) Multimaterial vertical 3D cryo(bio)printing of hydrogel filament array. (**F**) Vertical 3D cryo-bioprinting for fabricating the muscle-tendon unit. Reprinted with permission from [123], Copyright 2021, Wiley.

A more advanced strategy is to fuse different technologies to synthesize their advantages. For example, cryoprotective bioinks containing skeletal muscle cells were vertically extruded printed onto cryoplates to achieve macroscopic filamentous structures and microscopic gradient axial channels (Figure 8). Directional freezing overcomes the disadvantage of poor mechanical properties of bioinks in 3D printing, enabling the directional arrangement of cells at different levels [123]. Notably, although topology has a positive effect on cell adhesion and alignment, the addition of adhesion molecules is still necessary to enhance cell–macroporous hydrogel interactions [235,237,238].

## 4. Conclusions and Perspectives

Macroporous hydrogels are ideal for mimicking natural tissues due to their excellent diffusion of substances and infiltration of surrounding host cells. In recent years, great progress has been made in the preparation of macroporous hydrogels, which are mainly divided into sacrificial templates (including porogen templating, cryogel, Pickering emulsion, and gas foaming) and assembled templates (including 3D printing, electrospinning, granular hydrogels, and microribbons). If fabricated properly, macroporous hydrogels can mimic the properties of target tissues, such as pore size/shape/orientation, stiffness support, biochemical stimulation, and cell signaling. After encapsulating cells in vitro or implanting them in vivo, macroporous hydrogels regulate cell behavior according to their properties. A comprehensive understanding of the molecular mechanisms underlying the interaction of macroporous hydrogels with cells is beneficial to better mimic the native conditions of the ECM.

Current studies on macroporous hydrogels mainly focus on exploring the pluripotency of hydrogel precursors and topological advantages. Due to the risk of foreign body recognition and immune rejection in grafts, autologous biocomponents are an excellent choice for the host or modified macroporous hydrogels [242]. More autologous bioactive components need to be developed to incorporate macroporous hydrogels to mimic the target ECM more accurately in the future. Furthermore, tuning the pore shape, arrangement, and pore size gradient intuitively altered cell morphology and migration. The development of topologies that are more suitable for cellular behavior, such as anisotropic macropores and radial macropores, could further improve the effectiveness of macroporous hydrogels in tissue engineering [124,243].

However, applying macroporous hydrogels to the clinic needs to overcome several challenges. From a technical point of view, the large-scale use of macroporous hydrogels depends on the simplicity of the fabrication, use of steps, and the reproducibility of the results. Granular hydrogels (microfluidics) and 3D printing are considered to meet the above requirements based on the stability and ease of fabrication methods. However, granular hydrogels lack the complexity of their organization and lose their finer topology. Although 3D printing meets the above requirements, the high cost of high-precision 3D printers limits their application. Furthermore, functional cells and factors are an integral part of tissue engineering. The storage and mass production of them still require a lot of human labor and financial expenditure. Notably, most macroporous hydrogels lack clinical trials in humans. Hydrogel precursors and preparation methods need to undergo a rigorous review process to determine their long-term safety and efficacy, including material functionality, immunity, and service life.

In conclusion, this review discussed and highlighted the impact of the physicochemical properties of macroporous hydrogels in tissue engineering at the cellular biological level. It requires researchers from different disciplines to collaborate and utilize their complementary expertise to further advance the preparation and clinical translation of macroporous hydrogels, and more surprising advances in this field are expected in the coming years.

## Figures and Tables

**Table 1:** Overview of common techniques used to fabricate macroporous hydrogels and their advantages/disadvantages in tissue engineering.

Macroporous Hydrogels Preparation			Most Necessary Conditions	Advantages	Disadvantages	Polymer Examples	References
Sacrifice templating	Porogen templating		Porogens: Inert Compatible with gelation Fast and complete degradation	Easy processing Versatile Controllable pore size and shape (based on porogens design)	Resolution Limited architectures Poor pore connectivity Uncontrollable pore arrangement Cytotoxic solvents Difficult to completely remove porogen	Natural: Silk fibroin–cellulose Alginate Gum tragacanth	[38,39,40]
						Synthetic: Poly(2-hydroxyethyl methacrylate) Poly(NIPAAm-co-VP-co-MAPLA) Poly(ethylene-glycol)	[41,42,43]
						Natural/synthetic: Gelatin/poly(vinyl alcohol)	[44]
	Cryogel		Complete solution–gel transition at freezing temperatures	Easy processing Controllable pore size (based on the freezing temperature) Controllable pore arrangement (based on the direction of ice crystal formation)	Uncontrollable pore shape Low temperature impairs cell activity	Natural: Glycosaminoglycans Locust bean gum–xanthan gum–mastic gum Marine collagen-chitosan–fucoidan Gelatin Gum tragacanth Kefiran Chitosan–gelatin Hyaluronic acid Silk fibroin Platelet lysate–oxidized dextran Gelatin methacrylate–hyaluronic acid	[45,46,47,48,49,50,51,52,53,54,55,56]
						Synthetic: Polyurethane–calcium peroxide Polyethylene glycol	[57,58]
						Natural/synthetic: Chitosan/polyvinyl alcohol Poly(vinyl alcohol)/karaya gum Poly(vinyl alcohol)/carboxymethyl chitosan–dopamine Xanthan gum/poly(vinyl alcohol)	[59,60,61,62]
	Pickering emulsion		Emulsions: Inert emulsions Solvents able to miscible with each other Granular stabilizer: Two-phase wettability Suitable particle size Adjustable topography Suitable charge	Easy processing Scalability Good pore connectivity	Resolution Limited architectures Uncontrollable pore size, shape, and arrangement	Natural: Collagen Sodium alginate	[63,64]
						Synthetic: Poly(N-vinyl caprolactam) Polyacrylamide	[65,66]
						Natural/synthetic: Gelatine methacrylamide/poly(ethylene glycol)	[67]
	Gas foaming		Gel point is matched to the foam processes	Easy processing Inexpensive	Resolution Limited architectures Poor pore connectivity Uncontrollable pore size, shape, and arrangement Difficult to completely remove porogen	Natural: Gelatin–alginate Oxidized alginate–gelatin–silk fibroin	[68,69,70]
						Synthetic: Polycaprolactone Poly(ethylene glycol)diglycidyl ether	[71,72]
						Natural/synthetic: Gelatin-oxidized sodium alginate/adipic acid dihydrazide	[73]
Assembly templating	3D printing	Extrusion printing	Viscosity ranges from 6–30 × 10^7^ mPa s	Complex architectures Rapid prototyping Scalability Versatile	Limited to printable hydrogels Expensive Resolution	Collagen Methacrylamide-modified gelatin Decellularized extracellular matrix Poly(ε-caprolactone)	[74,75,76,77,78,79,80,81]
		Inkjet printing	Viscosity ranges from 3.5–12 mPa s			Poly-ɛ-lysine/gellan gum Alginate	[82,83]
		Stereolithography	No limitation			Methacrylamide-modified gelatin	[84,85]
		Laser-assisted printing	Viscosity ranges from 1–300 mPa s			Collagen	[86]
	Electrospinning		A sufficiently viscous polymer solution Relative humidity High pressure Collector Working distance Solution viscosity Flow rate Correct gel time	Resolution Rapid prototyping	Cytotoxic solvents Extensive post optimization	Natural: Alginate–gelatin Gelatin methacryloyl Keratin/chitosan Polysucrose	[87,88,89,90,91]
						Synthetic: Poly(oligoethylene glycol methacrylate) Poly(N-isopropylacrylamide-co-N-isopropylmethacrylamide) Poly(aspartic acid) Poly(ethylene glycol)	[92,93,94,95,96,97]
						Natural/synthetic: Chitosan/poly(vinyl alcohol)	[98]
	Granular hydrogels		Self-assembly Shear thinning Self-healing	Inexpensive Single-cell handling Reduced reagent consumption Controllable pore size and shape (based on granular design)	Non-standard cell culture Small volumes	Natural: Norbornene–hyaluronic acid Acrylamide–hyaluronic acid Gelatin methacryloyl	[99,100,101,102,103]
						Synthetic: PEDOT:PSS Poly(sulfobetaine methacrylate) Poly (sulfobetaine methacrylate)	[104,105,106]
						Natural/synthetic: Thiolated sodium alginate/hyperbranched poly (poly (ethylene glycol) diacrylate)	[107]
	Microribbons		High viscosity solvent to fix microstrip shape Secondary cross-linking assembly	Inexpensive Good pore connectivity	Resolution Limited architectures Extensive post optimization Cytotoxic solvents	Natural: Gelatin Chondroitin sulfate Hyaluronic acid	[108,109]
						Synthetic: Polyethylene glycol	[109]

**Table 2:** Anisotropic macroporous hydrogels in tissue engineering.

The Preparation of Anisotropic Macroporous Hydrogels		Hydrogel Polymers	Target Tissues/Cells	Effects	Reference
Sacrifice templating	Solid sacrificial template	Polyacrylamide–YIGSR peptide	Schwann cells	More spread Oriented morphology	[235]
		Polyacrylamide	Fibroblasts	Directional and faster migration	[236]
	Directional freezing	Agarose–gelatin	NIH-3T3	Directional migration	[209]
		Alginate	Bone marrow stromal cells	Differentiate into the neuron and glial cells	[237]
			Blood vessels	Increased vascular infiltration	
Assembly templating	Electrospinning	Alginate	hMSCs	Directional adhesion	[238]
		Polyacrylonitrile	ADSCs and cardiomyocytes	Directional adhesion and migration	[239]
		GelMA	Smooth muscle cells	Directional adhesion and migration	[212]
	3D printing	Alginate	ADSCs	Directional adhesion and migration	[240]
		GelMA	ADSCs	Directional adhesion and migration	[241]
			Chondrocytes	Directional adhesion and migration More ECM expression More proliferation	
Composite technology	3D printing-directional freezing	GelMA	Myoblasts and fibroblasts	Oriented morphology Muscle-tendon unit	[123]

## Data Availability

Not applicable.
